# 48-year-old with Coronavirus Disease 2019

**DOI:** 10.5811/cpcem.2020.4.47648

**Published:** 2020-05-07

**Authors:** Holly Gil, Ryan M. Finn, Neha P. Raukar

**Affiliations:** * Brown University, Department of Radiology, Providence, Rhode Island; † Mayo Clinic, Department of Emergency Medicine, Rochester, Minnesota

**Keywords:** COVID-19, imaging, Xray (radiograph), CT (computed tomography)

## Abstract

**Case Presentation:**

A 48-year-old male who presented with signs and symptoms suggestive of an upper respiratory infection was seen at an urgent care, he had a negative chest radiograph and was discharged. With no other cases of coronavirus disease 2019 (COVID-19) in the state, the patient presented to the emergency department two days later with worsening shortness of breath.

**Discussion:**

There are a variety of findings on both chest radiograph and computed tomography of the chest that suggests COVID-19.

## CASE PRESENTATION

A 48-year-old man with a history of asthma and reflux presented to the emergency department (ED) with a dry cough, sore throat, pleuritic chest pain, and dyspnea on exertion a week after serving as a tour guide in Europe and sharing equipment with other tour guides. He had been seen at an urgent care two days prior where he had a normal chest radiograph (CXR) and was discharged. On arrival to the ED, he was hemodynamically stable but had an oxygen saturation of 87% on room air, was tachypneic, using accessory muscles, and was febrile to 103.2° Fahrenheit. He was intubated secondary to respiratory distress. CXR and computed tomography (CT) were done in the ED, and it was later confirmed he was infected by severe acute respiratory syndrome coronavirus 2 (SARS-CoV-2),^1^ which causes coronavirus disease 2019 (COVID-19).^2^

**Image 1:**
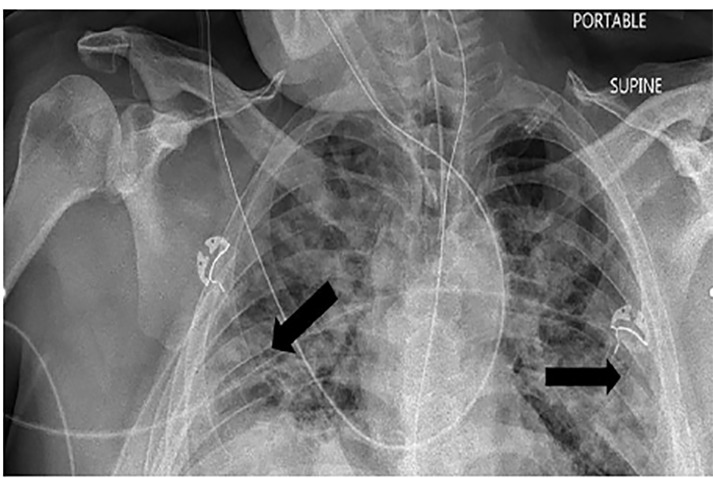
Chest radiograph with peripheral airspace opacities (arrows).

The primary finding on CXR is airspace opacities that are often bilateral or peripheral and found typically in the lower zones (Image 1).^3^,^4^

**Image 2:**
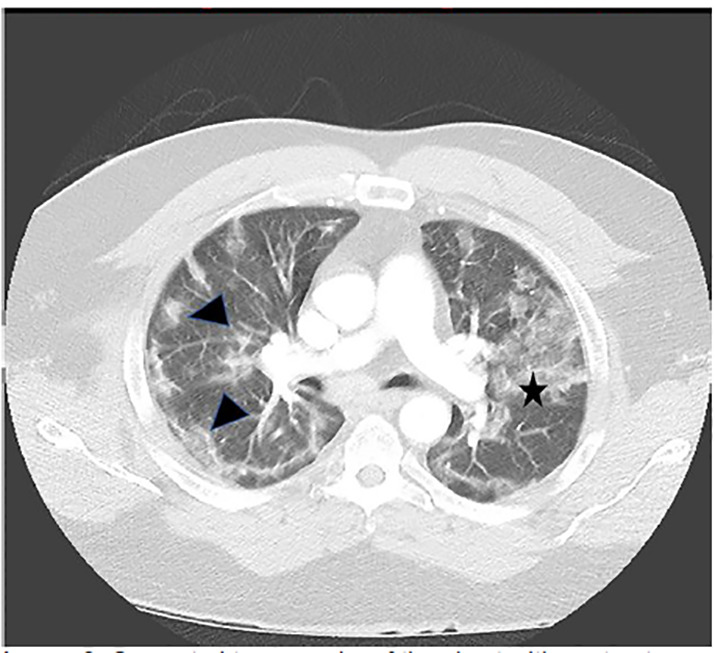
Computed tomography of the chest with contrast that demonstrates peripheral, ground-glass opacities (GGO) (arrowheads) in the periphery and a large area of GGO (star).

While there are over a dozen non-specific findings suggestive of COVID-19 on CT, those with the highest discriminatory values were ground-glass opacities (GGO), and GGO that are bilateral and/or peripheral in distribution (Image 2).^5^

## DISCUSSION

Given the infectious nature of SARS-CoV-2, a portable, single-view CXR is preferred to limit contamination.^6^ Of those hospitalized, CXR is abnormal 69% of the time and findings are most prominent 10–12 days after symptom onset.^4^

Within the first two days of symptom onset, CT is normal 56% of the time, and after day three of symptoms is abnormal in at least 90% of patients.^7^ Despite the non-specific nature of these findings, radiologists are able to distinguish between COVID-19 and viral pneumonia with high specificity and moderate sensitivity.^5^ Although not diagnostic, imaging can suggest the presence of COVID-19 disease, and the American College of Radiology has adopted standardized language to reduce reporting variability.^8^

**CPC-EM Capsule**

What do we already know about this clinical entity?

The high infectious state, especially when asymptomatic, and increased mortality seen with severe acute respiratory syndrome coronavirus 2 has led to a global pandemic.

What is the major impact of the image(s)?

Bilateral and/or peripheral airspace opacities on radiographs and computed tomography can help suggest infection before testing results are available.

How might this improve emergency medicine practice?

Early identification of potentially positive cases can help the healthcare team maintain vigilance in protecting themselves and when indicated and available, start treatment early.
